# Revisiting the co-existence of Attention-Deficit/Hyperactivity Disorder and Chronic Tic Disorder in childhood—The case of colour discrimination, sustained attention and interference control

**DOI:** 10.1371/journal.pone.0178866

**Published:** 2017-06-08

**Authors:** Henrik Uebel-von Sandersleben, Björn Albrecht, Aribert Rothenberger, Anke Fillmer-Heise, Veit Roessner, Joseph Sergeant, Rosemary Tannock, Tobias Banaschewski

**Affiliations:** 1 Child and Adolescent Psychiatry, University Medical Center Göttingen, Göttingen, Germany; 2 Department of Child and Adolescent Psychiatry, Technische Universität Dresden, Dresden, Germany; 3 Department of Clinical Neuropsychology, Faculteit der Psychologie, Vrije University, Amsterdam, The Netherlands; 4 The Hospital for Sick Children, Toronto, Canada; 5 Department of Child and Adolescent Psychiatry and Psychotherapy, Central Institute of Mental Health Mannheim, Mannheim, Germany; University of Melbourne, AUSTRALIA

## Abstract

**Objective:**

Attention Deficit / Hyperactivity Disorder (ADHD) and Chronic Tic Disorder (CTD) are two common and frequently co-existing disorders, probably following an additive model. But this is not yet clear for the basic sensory function of colour processing sensitive to dopaminergic functioning in the retina and higher cognitive functions like attention and interference control. The latter two reflect important aspects for psychoeducation and behavioural treatment approaches.

**Methods:**

Colour discrimination using the Farnsworth-Munsell 100-hue Test, sustained attention during the Frankfurt Attention Inventory (FAIR), and interference liability during Colour- and Counting-Stroop-Tests were assessed to further clarify the cognitive profile of the co-existence of ADHD and CTD. Altogether 69 children were classified into four groups: ADHD (N = 14), CTD (N = 20), ADHD+CTD (N = 20) and healthy Controls (N = 15) and compared in cognitive functioning in a 2×2-factorial statistical model.

**Results:**

Difficulties with colour discrimination were associated with both ADHD and CTD factors following an additive model, but in ADHD these difficulties tended to be more pronounced on the blue-yellow axis. Attention problems were characteristic for ADHD but not CTD. Interference load was significant in both Colour- and Counting-Stroop-Tests and unrelated to colour discrimination. Compared to Controls, interference load in the Colour-Stroop was higher in pure ADHD and in pure CTD, but not in ADHD+CTD, following a sub-additive model. In contrast, interference load in the Counting-Stroop did not reveal ADHD or CTD effects.

**Conclusion:**

The co-existence of ADHD and CTD is characterized by additive as well as sub-additive performance impairments, suggesting that their co-existence may show simple additive characteristics of both disorders or a more complex interaction, depending on demand. The equivocal findings on interference control may indicate limited validity of the Stroop-Paradigm for clinical assessments.

## Introduction

Attention Deficit / Hyperactivity Disorder (ADHD) and Chronic Tic Disorder (CTD) are considered as neurodevelopmental disorders DSM-V [1], and both associated with dopaminergic dysfunction. Differential characterisation of related cognitive functions may clarify pathophysiological underpinnings in light of their co-existence, and thus may give new insights for diagnosis, psychoeducation and treatment.

In **ADHD** dysfunctions in dopaminergic fronto-striatal neuronal networks may play an important role in the development and retention of problems with inhibitory executive functions or cognitive control as one of its core deficits [2]. Deficits in sustained attention, preparation, and cognitive control are often demonstrated at the level of performance and associated brain activity [3].

**Tic disorders** are probably associated with disturbances in cortico-striato-thalamico-cortical neuronal networks, which may be partly compensated by increased prefrontal activity incorporated in tic suppression. A recent publication on response inhibition during Stop-Task performance highlighted the role of possible compensatory mechanisms: adults with CTD showed no performance deficits, but activity in the dorsal premotor area during successful Stops was lower than in controls, and elevated activity in the supplementary motor area was related to tic frequency [4].

**Co-existence of ADHD+CTD** is much more frequent than expected from the product of both disorders’ prevalence [5, 6], and the reasons for this are still not well understood. There seem to exist no clear performance deficits in CTD regarding tasks tapping executive functions, and studies addressing the co-existence of ADHD+CTD found deficits explained by the ADHD factor following an additive model [7–10]. In contrast, studies on brain activity during preparation and self-regulation may somewhat challenge the additive model of ADHD+CTD comorbidity [11, 12]. In sum, it is suggested that ADHD+CTD may follow an additive model for basic sensorymotor aspects and probably a merely non-additive model if higher functions and more complex tasks come into play [13, 14]. Hence, further investigation of cognitive abilities are warranted to elucidate which model of ADHD + CTD (additive/interactive) would best explain its co-existence.

The aim of the current study was to test the additive model of ADHD+CTD co-existence by investigating performance of colour perception and two measures of higher order executive functions using a controlled four-group design (ADHD, CTD, ADHD+CTD, Controls).

**Colour perception** is of particular importance in everyday life. According to the retinal dopaminergic hypothesis, deficient dopaminergic signalling in the central nervous system may be reflected in colour perception deficits particularly in the short wavelength S-cones which are rare and particularly sensitive to dopamine [15]. There is growing evidence that colour vision particularly on the blue-yellow axis may be impaired in children and adults with ADHD [16, 17] and CTD [18]—we refer to these data again, as colour discrimination is implicated in the Colour-Stroop and may have an impact on interference load.

**Sustained attention** refers to the ability to stay focussed on a task and resist distraction over longer periods of time has been linked to brain functions located in the prefrontal and parietal cortex [19]. Sustained attention is thus an implicit part of many cognitive tasks e.g. like the Continuous Performance Test, and individuals with ADHD, in particular, show diminished performance and lower brain activity during cue processing and preparation [20].

**Interference control** comes into play when conflicting task demands are faced or distraction needs to be restrained. It has been regarded as one of the core deficits in ADHD [21]. It may be assayed with the Stroop-Test. The general idea is that processing of the target is less automatized and slower than distractor processing, and so requires effortful control if the target and distracting information is incongruent. This was demonstrated for naming the colour vs. reading a colour-word as early as 1886 by McKeen Cattell and in 1935 it was utilized by Stroop in his classic interference task (for a review see [22]). The task yields relative robust interference effects, but this is an epi-phenomenon, arising from the requirement to name the colour of the targets in neutral compared to incongruent conditions. In the latter condition, automaticity in reading the colour word interferes with naming the colour in which the word is printed. It has been demonstrated that relative speed or automaticity of reading colour words compared to colour naming is crucial, such that excessive training in colour naming can lead to a reverse Stroop effect [22].

Interference control during Stroop-Task performance has been assessed frequently in ADHD, but the interpretation of findings is still incomplete. Recent meta-analyses on standard implementations and analyses of the Colour-Stroop indicated no significantly larger interference liability in children or adults with ADHD [23, 24]. However, as pointed out by Lansbergen et al., this may hold particularly for studies quantifying the interference effect considering word reading speed [25], whilst a meta-analysis on studies calculating the simple difference score between neutral and incongruent items revealed significantly larger interference liability in ADHD [26].

Only a few studies have assessed interference liability in CTD. The results suggest no deficient performance in CTD [10, 27], but studies on brain activity revealed increased brain activity associated with self-regulation during Stroop performance that may indicate successful compensatory mechanisms in CTD [28].

Besides these inconclusive findings, the validity of the Colour-Stroop may be limited if colour perception deficits come into play, as this may hamper processing of the target information and may thus lead to larger interference effects. For the current study, we therefore employed also a Counting-Stroop as described by [29], where quantities of up to four items (neutral dots or incongruent numbers) need to be perceived rapidly and accurately at a glance, without counting (i. e. subitized). As suggested by a recent study in children, digit naming may be faster than subitizing [30], supporting that the Counting-Stroop may be in principle an ideal parallel version of the classical Colour-Stroop for that age range.

### Hypotheses

A previous study on an almost identical sample showed that both disorders are associated with colour discrimination deficits, following an additive model, which are for ADHD particularly expressed on the blue-yellow axis [18]. Since colour discrimination deficits may diminish the validity of the Colour-Stroop, a parallel Counting-Stroop is used to explore whether suspected effects on interference liability are also present if uncoloured quantities are subitized in Stroop-like manner.

It is hypothesized that attention deficits are eminent in ADHD, but not in CTD (i.e., effect size for CTD will be smaller than that for ADHD and non-significant), and thus for attention support for an additive model is expected. Difficulties with Interference control (regarding response-speed or accuracy) in the Stroop-Tests are predicted to occur for ADHD but not CTD, since children with CTD and ADHD + CTD respectively might use mechanisms active during tic suppression to ameliorate deficits, possibly leading to sub-additive effects in ADHD+CTD.

## Material and methods

### Subjects

A total of 73 subjects aged 8 to 13 years participated in the study on the basis of written informed consent from child and parent and approval from the ethics committee of the University Medical Center Göttingen. All subjects were free of ophthalmologic disorders or congenital colour blindness and had a full-scale IQ of at least 85, normal or corrected to normal vision and understood task instructions as verified by practice trials. Participants belonged to a group of healthy Controls (N = 15, 2 ♀) or were diagnosed according to ICD-10 with attention deficit/hyperactivity disorder combined type (ADHD, also according to DSM-IV, N = 14, 1 ♀)), with CTD (TIC, N = 20, 2 ♀)), or with comorbid ADHD+CTD (N = 20, 4 ♀); due to gender and age matching, 2 participants with ADHD and 2 Controls were excluded.

Due to the group matching, there were no differences in gender-ratio (both χ^2^_(1)_<0.4, p>.57) and age between children with and without ADHD or CTD (all (F_(1, 65)_<1, p>.7, part η^2^ <.01, see Table 1 for further details). Children with ADHD showed a significantly lower prorated mean IQ (F_(1, 65)_ = 7.8, p <.01, part η^2^ = .11, see table), whilst spelling abilities and word fluency did not differ between groups. Dyslexia was present in 8 of the 69 participants (with missing diagnostic data for one subject, see Table A in S1 File), but the proportion did not differ between the four groups (χ^2^_(1)_ = 2.1, p = .55).

**Table 1:** Sample description.

Measure	Controls (C)	ADHD (A)	CTD (T)	ADHD+CTD (AT)	ANOVA F_(1,65)_		
	Mean (SD)	Mean (SD)			ADHD	CTD	ADHD*CTD
**Sample Size (♀)** ^a^	15 (2)	14 (1)	20 (2)	20 (4)	χ^2^_(1)_ = 0.16	χ^2^_(1)_ = 0.32	χ^2^_(1)_ = 1.1
**Age (in months)**	129 (10.0)	128 (11.3)	127 (15.8)	128 (14.8)	<1	<1	<1
**Prorated-IQ**	113 (13.6)	103 (7.9)	108 (10.7)	103 (9.0)	7.8** (.11)	1.1 (.02)	1.4 (.02)
**SDQ parents-rated** ^b^							
Hyperactivity	1.3 (1.4)	8.2 (2.0)	4.0 (2.1)	6.7 (2.5)	86.9** (.58)	1.4 (.02)	15.3** (.20)
Prosocial Behaviour	8.3 (1.7)	6.4 (2.1)	7.3 (1.9)	7.1 (1.7)	5.0* (.07)	<1	3.7+ (.06)
Emotional Symptoms	0.9 (1.3)	3.2 (1.7)	3.0 (2.6)	4.0 (3.0)	7.6** (.11)	6.2* (.09)	1.3 (.02)
Conduct Problems	0.7 (0.7)	4.9 (2.1)	1.7 (1.5)	3.3 (1.9)	52.7** (.46)	<1	9.8** (.13)
Peer Problems	0.7 (1.1)	3.3 (2.6)	1.3 (1.8)	3.6 (2.7)	21.0** (.25)	<1	<1
Total	3.6 (3.0)	19.6 (6.6)	9.9 (5.7)	17.6 (8.0)	59.7** (.49)	1.9 (.03)	7.4* (.11)

^+^ p <.1

* p <.05

** p <.01

^a^ Gender-ratio tested for factors ADHD, CTD and ADHD*TIC (Controls and ADHD+TIC vs. ADHD and TIC) with χ^2^_(1)_-tests

^b^ SDQ parents missing in one child with ADHD and one with ADHD+TIC, thus F_(1,63)_.

Control children were recruited from local schools and never met a child psychiatric disorder except dyslexia, and T-scores of the CBCL scales for attention problems were required to be below 55 as well as delinquent and aggressive behaviour below 60. Further details about diagnostics and the data on colour perception in ADHD and CTD are provided in a previous publication [18].

Patients had been recruited from sequential referrals of the outpatient clinic of the Department of Child and Adolescent Psychiatry at the University of Göttingen. Diagnosis was based on information obtained from clinical assessment by a board certified child psychiatrist including interviews with the parents and child, as well as teacher reports and behaviour rating scales including parent-rated Child Behavior Checklist (CBCL, [31, 32]), Strengths and Difficulties Questionnaire (SDQ, [33, 34]) and the German version of the ADHD symptom list (FBB-HKS, [35]). Children with TIC or ADHD+TIC were additionally assessed with the Yale Tourette Syndrome Symptom List [36] and the Tourette Syndrome Severity Scale [37]. Those children using methylphenidate were free of medication for at least 48h before testing, while medication with D2-blockers (CTD: N = 2/20; ADHD+CTD: N = 7/20), serotonin reuptake inhibitors (N = 1) and atomoxetine (n = 3) were continued.

The available total sample of 69 participants permits the detection of large effects (η^2^>10.5% explained variance) with 80% power (1-β) and α set to 5%; considering also trends (α<10%) highlight almost medium effects (η^2^>8.4%).

### Tasks and procedure

The test session was carried out under standardized light conditions (using white tubular fluorescent daylight lamps at 325 lux, measured with a LT Lutron LX-101 Lux-meter, which is in accordance with the manual of the colour discrimination test) in a noise-shielded room. Besides the main parameters for the current study, children also underwent standardized IQ-testing, tests of Spelling Abilities and Word Fluency ([38], see Table A in S1 File) and a classical “off-line” Stroop-Test [39] using cards each with 72 congruent, incongruent, or neutral items (results were presented previously by [18]).

**Colour discrimination** as measured by the Farnsworth-Munsell 100 hue Test is frequently used in ophthalmological investigations [40]. The test requires ordering the colour hue of round plates on four rows regarding orange-magenta, yellow-green, blue-purple and purple-magenta. Performance is characterised by error scores for the blue-yellow and red-green axis as well as a total error score. Conducted under standard light conditions, the test may has acceptable validity and internal consistency for group comparisons [41], but colour discrimination performance may also depend on motivation, comprehension, skill [42] and possibly nonverbal IQ [43].

**Sustained attention** was assessed with the Frankfurt Attention Inventory (FAIR). This 6-minute paper-and-pencil test requires marking two prespecified targets in a row of items with a line drawn continuously along each row. An example of the test procedure and a more detailed description is given in the Figure A in S1 File. Performance is quantified in the main parameters L (indicating the error-corrected estimate of concentrated processed number of items as an indicator of processing speed), Q (quality or accurateness, as the proportion of L amongst the total number of processed items) and K (the product of Q*L as a continuity indicator) [44]. In addition, we analysed also the false-alarms rate as this may be a sensitive parameter for an impulsive response style.

**Fig 1:**
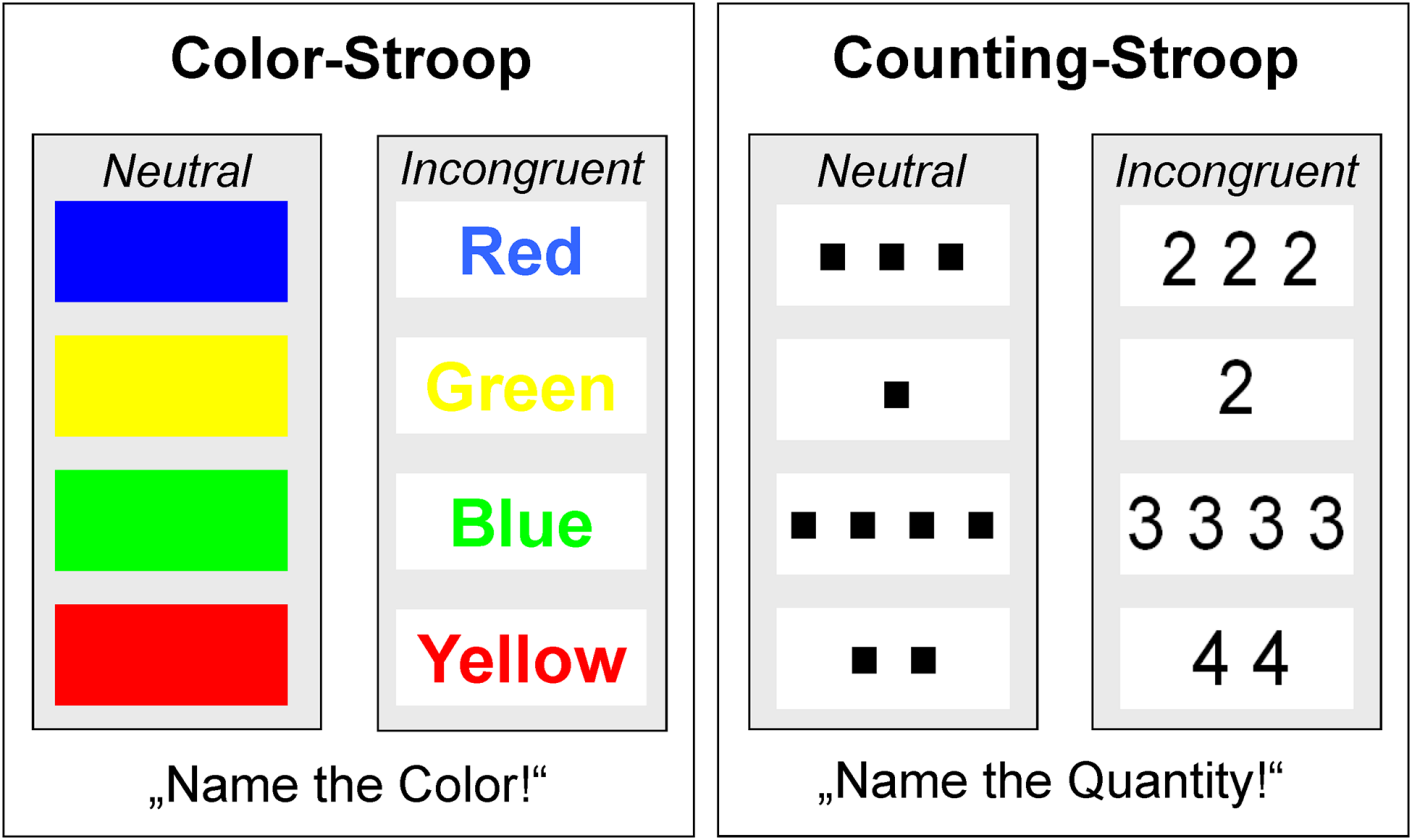
Stroop-Task description. Items of Colour- and Counting-Stroop. Correct responses are “blue”, “yellow”, “green” and “red” for the Colour-Stroop and “3, “1”, “4” and “2” for the Counting-Stroop. Responses were given on a custom-made trapezoid four-choice response pad.

**Interference Control** was assessed with two conditions of a computerized single trial Stroop-Task: one condition used colours as targets (coloured bars or colour words printed in incongruent colours); and the other used quantities (squares or numbers printed in black) used as targets presented in neutral or incongruent stimuli (Fig 1). Each configuration was presented randomized in a block-wise design. Each block started with written instructions and practice trials as required for understanding the task, followed by 72 experimental trials. The targets were presented in the centre of a 17” CRT monitor against a light grey background at a viewing-angle of approx. 3° horizontally and 1° vertically. Each trial started with the presentation of a fixation mark for 250ms, a blank screen for another 250ms and the presentation of the stimulus for 750ms followed by a blank screen for 1000ms. The participants had to respond with the index finger or thumb of both hands using a four choice response box assigned to colours (button layout was an isosceles trapezium; from left to right, upper and lower row: green, red, blue and yellow) or quantities (1, 2, 3 and 4, see Figure B in S1 File). The instructions emphasized speed and accuracy equally.

Total task duration including three supplementary conditions not analysed here was 30 min. Reaction time (RT), intra-individual reaction-time variability (RT-SD) and response accuracy (percentage of correct responses) were recorded for each condition.

### Analyses

The dependent variables were tested in a 2×2 factorial design with the between subject factors “ADHD” (children with vs. without ADHD) and “CTD” (children with vs. without CTD disorder). In the Farnsworth-Munsell 100hue Test, error-scores on the red-green and blue-yellow axes were analysed as repeated measures on within-subject factor “Axis” (see Table A in S1 File). For the single-trial Stroop-Test, trials with reaction-times faster than 150ms were discarded, and data from the four response buttons were collapsed into a grand mean (see Table B S1 File). Dependent variables from the two single-trial Stroop tasks were analysed with repeated measure analysis of variance (ANOVA), in which “Condition” (colour-naming vs. counting) and “Congruency” (neutral vs. incongruent stimuli) were within subject factors, and “ADHD” and “CTD” were between subject factors. In case of interaction effects, additional post hoc analyses of the confidence intervals with p = .05 were performed. Since the groups differed in IQ, the analyses were also conducted with IQ taken as covariate. Effect sizes were computed using partial η^2^ with part. η^2^>.01 considered as small, part. η^2^>.06 as medium and part. η^2^>.14 as large effects [45].

Inter-individual speed-accuracy trade-offs were analysed as repeated measure factor with z-transformed reaction times of correct responses (multiplied by -1, such that higher values indicate faster response speed) and z-transformed numbers of correct responses (thus, higher values indicate higher performance in both domains). Main effects would thus indicate general performance differences; interactions with the speed-accuracy factor would indicate relative shifts in the trade-off.

The influence of colour perception was explored as following: since error scores on the blue-yellow and red-green axes are highly correlated with the total error score in the overall sample (both r_(69)_>.92, p <.01), this total error score in addition with IQ (to control for effects of general cognitive ability) and age (to control for developmental effects) were used as covariates in the general linear model of the speed-accuracy assessment, including the main and within-subject interaction effects with the covariates.

Testing the additive model of ADHD and CTD on colour discrimination, sustained attention and interference liability requires adjustments for multiple comparisons, such that three type one errors may accumulate in directed testings, requiring an adjusted nominal α = .05*2(one-tailed testing)/3(number of hypotheses) = .033.

## Results

In the following section we present the results of the 2×2 factorial design. If not otherwise stated, ADHD and CTD are thus regarded as factors. E.g. ADHD denotes children with pure ADHD and ADHD+CTD in comparison with Controls and pure CTD, whilst the CTD factor comprises the comparison of pure CTD and ADHD+CTD in comparison with Controls and pure ADHD groups. In case of significant interaction effects between ADHD and CTD, the four groups were compared in post-hoc tests to further clarify violations of the additive model of ADHD and CTD co-occurrence.

### Psychopathology

The parent-rated SDQ revealed problems in children with ADHD for all scales (ADHD: all (F_(1, 65)_>5.0, p <.03, part η^2^>.07), but problems regarding Hyperactivity, Conduct Problems and Total Problems were particularly present in the ADHD-only group (ADHD*CTD: all F_(1, 65)_> 7.4, p <.01, part η^2^>.11). Children with CTD were rated with higher scores for Emotional Symptoms (CTD: (F_(1, 65)_ = 6.2, p = .02, part η^2^ = .09) while other comparisons revealed no differences (all other (F_(1, 65)_<2.0, p>.17, part η^2^ <.03, see Table 1).

### Colour discrimination: Farnsworth-Munsell 100 hue Test

**Fig 2:**
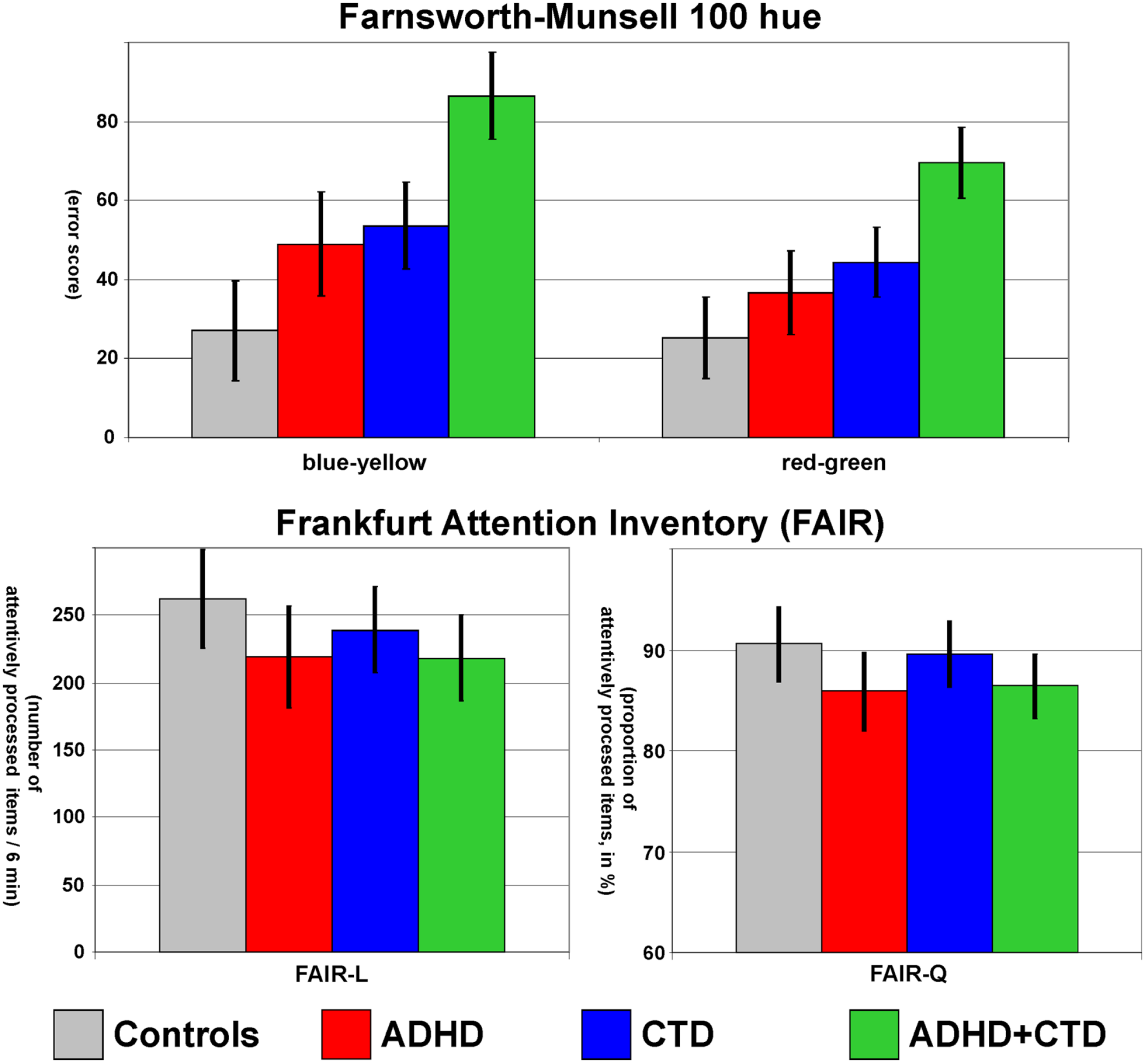
Colour discrimination and attention. The comorbidity of ADHD+CTD is characterized by additive effects on colour discrimination and attention. The Farnsworth-Munsell 100 hue colour discrimination test (above) revealed for ADHD and CTD additive effects on error scores (confidence intervals with p = .05) of the blue-yellow (left) and red-green (right) axis. Difficulties with sustained attention in the Frankfurt Attention Inventory as indicated by the number (FAIR-L, below left, confidence intervals with p = .05) and proportion (FAIR-Q, below right) of attentively processed items during the 6 min testing are present in children with ADHD but not CTD.

As reported previously [18], colour discrimination difficulties present in ADHD (F_(1, 65)_ = 22.6, p <.01, part η^2^ = .26) and CTD (F_(1, 65)_ = 36.5, p <.01, part η^2^ = .36) were additive (ADHD*CTD: F_(1, 65)_ = 1.7, p = .20, part η^2^ <.03, see Fig 2). Moreover, these difficulties were particularly expressed on the blue-yellow axis (Axis: F_(1, 65)_ = 15.2, p <.01, part η^2^ = .19), where children with ADHD tended to have more pronounced difficulties discriminating blue-yellow (ADHD*Axis: F_(1, 65)_ = 3.1, p = .08, part η^2^ = .05, but impairments in ADHD were significant on both axes & CDT*Axis: F_(1, 65)_ = 1.4, p = .25, part η^2^ = .02, see Table A in S1 File). Importantly, post hoc tests indicate elevated blue-yellow difficulties in all three clinical groups (all p <.05, in pure CTD p <.06), but not in controls (p>.75). These effects remained stable, when controlling for IQ.

### Sustained attention: Frankfurt Attention Inventory (FAIR)

Attention was measured with the FAIR including the performance parameter L (number of concentrated processed items during the 6 min assessment), quality (proportion of concentrated processed items, Q) and continuity (K). Children with ADHD showed diminished attention on all three parameters (as a trend on “L”, F_(1, 65)_ = 3.5, p = .07, part η^2^ = .05, but significant for “Q” and “K”, both F_(1, 65)_>4.7, p< = .03, part η^2^ = .07, see Table A in S1 File and Fig 2 above). In addition, children with ADHD made more false alarms during the FAIR (F_(1, 65)_ = 4.2, p = .05, part η^2^ = .06). However, with the exception of false alarms, these ADHD effects disappeared (L, K) or were diminished to a trend (Q) when controlling for IQ.

### Interference liability: Single-trial Stroop-Test

#### Reaction-time

**Fig 3:**
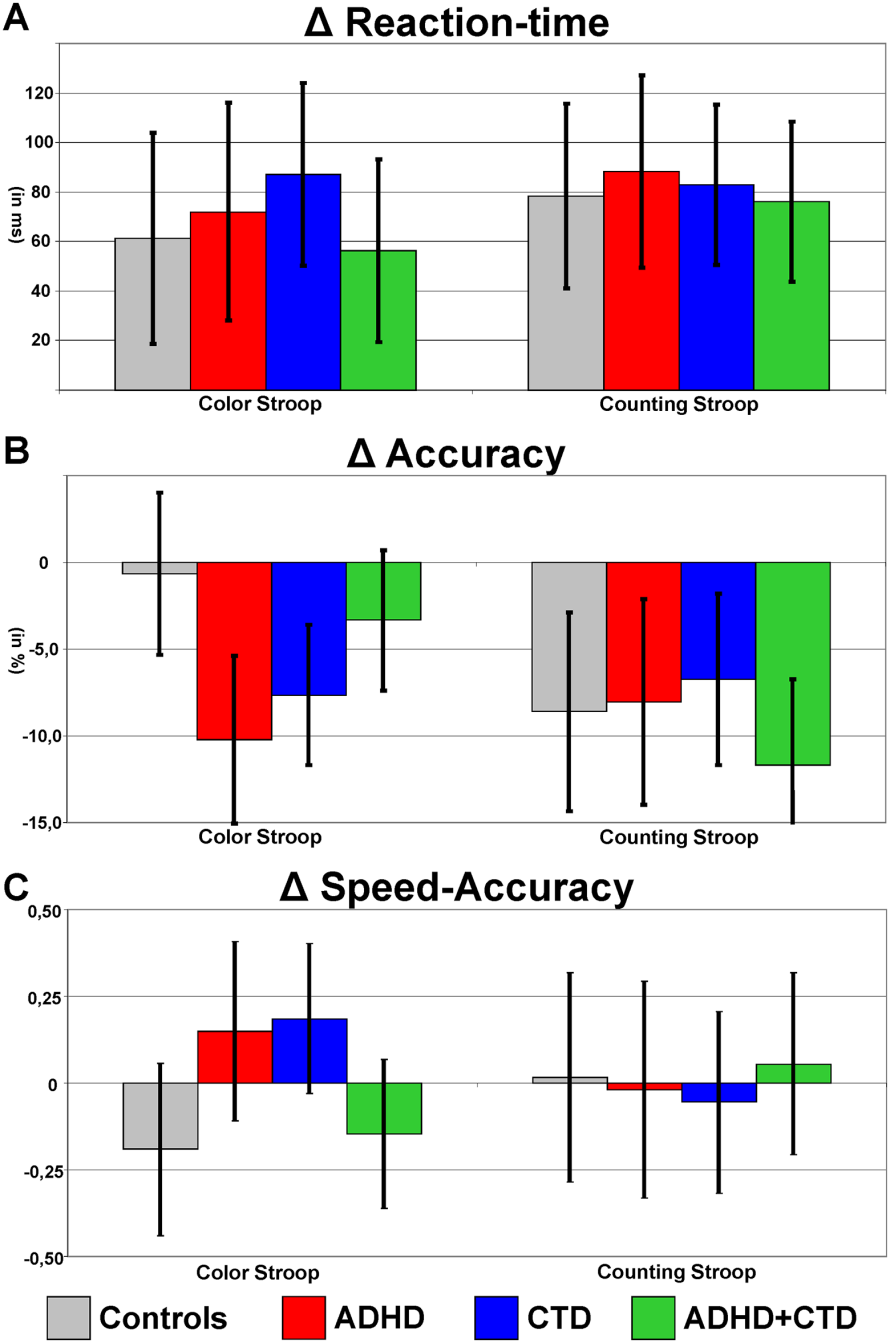
Congruency effects in the single-trial Stroop tasks. This Figure shows confidence intervals (p = .05) of the Congruency effects (as the respective differences in performance parameters between incongruent minus congruent conditions) from Colour- (left) and Counting-Stroop (right). Reaction-times were significantly slower in incongruent trials of both Stroop-Tests in all four groups (as the respective confidence intervals indicated by vertical bars around the respective mean do not include zero), and did not reveal any group-differences (as no mean lie outside the confidence intervals of comparison, see line A, above). Accuracy was significantly lower in incongruent trials of the Counting Stroop similarly for all groups (different from zero and negative in all groups, and all confidence intervals overlap with the respective means), but in the Colour Stroop interference liability on accuracy was present only in the pure ADHD and CTD groups, while Controls and children with ADHD+TIC did not show reduced accuracy in incongruent trials and showed less congruency effect as compared to both other groups (line B). The combined Speed-Accuracy parameter (line C, mean difference incongruent minus congruent trials of z-standardized reaction-time and accuracy scores) showed in the Colour Stroop (left) elevated interference load in the pure ADHD and pure CTD groups than in the controls and comorbid ADHD+TIC groups only, but no significant group differences in the Counting-Stroop (right).

Overall, reaction-times were similar for both Stroop-Tests (Condition: F_(1, 65)_<1, p = .93, part η^2^ <.01), and slower in incongruent compared to congruent trials (Congruency: F_(1, 65)_ = 115.6, p <.01, part η^2^ = .64). A significant main effect CTD (F_(1, 65)_ = 7.2, p = .01, part η^2^ = .10) and an interaction CTD*Condition (F_(1, 65)_ = 4.6, p = .04, part η^2^ = .07) were further clarified with post hoc analyses of confidence intervals: children with CTD showed slower RTs in both conditions (p <.05), and tended to be slower in the colour Stroop (p <.10) while children without tics showed rather (but not significantly, p <.20) the opposite pattern. No significant main effects or interactions with ADHD, and no interactions with Congruency were found (all F_(1, 65)_<1.1, p>.30, part η^2^ <.02, see Table B in S1 File). Confidence intervals of the Congruency-effect marginal means are given in Fig 3A. Controlling for IQ did not change these results.

#### Accuracy

Accuracy was similar in both Stroop conditions (Condition: F_(1, 65)_<1, p = .94, part η^2^ <.01), but lower in incongruent trials (Congruency: F_(1, 65)_ = 75.3, p <.01, part η^2^ = .54). However, besides the interaction CTD*Condition (F_(1, 65)_ = 7.5, p <.01, part η^2^ = .10), there was also a three-way interaction Condition*ADHD*CTD (F_(1, 65)_ = 14.8, p <.01, part η^2^ = .19) was found. Moreover, there were also two- and four-way interactions involving Condition and Congruency (Condition*Congruency: F_(1, 65)_ = 3.2, p = .08, part η^2^ = .05 and Condition*Congruency*ADHD*CTD: F_(1, 65)_ = 7.0, p = .01, part η^2^ = .10): Post-hoc analyses separately for each Condition revealed significant Congruency effects in both Stroops (both Intercept F_(1, 65)_>24.5, p <.01, part η^2^>.27) without any group differences in the Counting Stroop (all F_(1, 65)_<1.1, p>.31, part η^2^ <.02), but for the classical Colour Stroop an interaction ADHD*TIC (F_(1, 65)_ = 9.9, p <.01, part η^2^ = .13, see Table B in S1 File & Fig 3B) indicated significant congruency effects on accuracy in the pure ADHD and CTD groups but not in controls and children with comorbid ADHD+CTD. These results were unrelated to, and did not change when controlling for IQ.

#### Speed-accuracy

For the performance analysis taking speed and accuracy as factors, the parameters were at first z-standardized separately for congruent and incongruent trials of each of the two single trial Stroop-Tests. Therefore, no main effects of the within-subject factors Speed-Accuracy (S_A), Condition and Interference remained in this comparison (all F_(1, 65)_<1). Children with ADHD achieved lower speed-accuracy performance (F_(1, 65)_ = 11.3, p <.01, part η^2^ = .15) (which was correlated with colour discrimination problems, see below) whilst CTD and the interaction ADHD*CTD had no effect on general speed-accuracy in the Stroop-Tests (both F_(1, 65)_<1.5, p>.23, part η^2^ <.02).

Several interactions including speed-accuracy (S_A) reached significance: S_A*CTD (F_(1, 65)_ = 8.3, p <.01, part η^2^ = .11), S_A*Condition*CTD (F_(1, 65)_ = 9.7, p <.01, part η^2^ = .13) and S_A*Condition*CTD*ADHD (F_(1, 65)_ = 7.3, p <.01, part η^2^ = .10). Separate analyses of the difference in the z-standardized reaction-times and accuracy rates revealed differences between the two tasks: for the Colour-Stroop, a main effect of CTD (F_(1, 65)_ = 15.0, p <.01, part η^2^ = .19) and an Interaction ADHD*CTD (F_(1, 65)_ = 4.3, p = .04, part η^2^ = .06) was present. Post-hoc tests showed that in the pure ADHD group speed was emphasized over accuracy (the difference in z-values was significantly positive) whilst in children with ADHD+CTD and as a tendency also in children with pure CTD disorder (p <.10) accuracy was accentuated. For the Counting-Stroop, no group differences in speed-accuracy trade-off were found (all F_(1, 65)_<2.2, p>.14, part η^2^ <.03, see Figure C in S1 File).

Moreover, there was a fourth-order interaction Condition*Congruency*CTD*ADHD (F_(1, 65)_ = 5.0, p = .03, part η^2^ = .07) which was further explored with the Congruency differences, separately for both Stroop-Tests. In accordance with the separate analyses of response speed and accuracy, the congruency effect in the Colour Stroop was larger in children with pure ADHD and pure CTD, and smallest in controls and children with comorbid ADHD+CTD, whilst in the Counting Stroop the Congruency effects on speed and accuracy combined did not differ between groups (see Fig 3C for the respective congruency differences (congruent minus incongruent trials) in mean z-scores (the mean of z-reaction-time*-1 and z-accuracy) of both Stroop-Tasks).

#### The influence of colour discrimination on speed-accuracy Stroop performance

The following analysis is based on the speed-accuracy analysis, with age, IQ and Farnsworth-Munsell total error score (combined error score from both axes) initially entered as covariates; IQ was then dropped since it had no significant impact.

This covariance analysis revealed better combined speed-accuracy performance in older children (Age: F_(1, 63)_ = 8.5, p = .01, part. η^2^ = .12) and in children with better colour discrimination (F_(1, 63)_ = 6.1, p = .02, part. η^2^ = .09), as well a shift towards speed with ageing (S_A*Age: F_(1, 63)_ = 3.6, p = .06, part. η^2^ = .05 & S_A: F_(1, 63)_ = 3.2, p = .08, part. η^2^ = .05 –this is mainly due to faster RT, whilst accuracy remains unchanged with age). Importantly, total error score did not interact with the Congruency effect (all F_(1, 63)_<1.2, p>.28). Moreover, with the exception of the strong ADHD main effect (which is diminished to a trend when colour discrimination performance was controlled for, F_(1, 63)_ = 3.6, p = .06, part. η^2^ = .05), all the significant effects from the speed-accuracy analysis above were replicated with Age and colour discrimination total error score covaried.

## Discussion

Co-existence of disorders is the rule than the exception in the daily practice of child and adolescent psychiatry. ADHD and CTD are common and frequently co-existing posing many challenges for the clinician [14]. In the current study, we assessed children and adolescents with pure ADHD and pure CTD, co-existing ADHD+CTD and healthy Controls in a 2 by 2 statistical comparison for testing the additive model of co-existence.

### Sample characteristics

The sample of children (N = 69 subdivided in to patients with pure ADHD and pure CTD, comorbid ADHD+CTD and controls) permitted the detection of large effect sizes with a conventional α< 5% and a power of 80%, and close to medium effect size if trends α< 10% were also considered. Since impairments in ADHD are expected to be large, the current sample is adequate for detection, but as CTD and potential (sub-additive) ADHD*CTD interaction effects may be smaller, potential relevant effects may remain undetected even if trends were considered.

The gender ratio was successfully matched, thus assessment of possible gender effects were beyond the scope of the current study, and conclusions can only be drawn regarding clinical referrals, which are predominantly boys.

Psychopathological ratings obtained with the parent-rated SDQ revealed broad difficulties for children with ADHD. Children with CTD showed higher ratings of emotional problems, following an additive model. Sub-additive effects on Hyperactivity, Conduct Problems and also Total Problems may indicate that in children with co-existing ADHD+CTD psychopathological difficulties may follow ADHD, but may rather be compensated by the co-existing CTD [46]. This may supplement previous psychopathology findings using the parent-rated CBCL in a larger sample, where children with ADHD were also characterized by broad difficulties, but co-existence was mainly driven by additive effects, particularly regarding externalizing problems following ADHD and in addition rather internalizing problems also from CTD [47].

### Colour discrimination

Colour discrimination impairments of large effect size are present in ADHD and CTD following an additive model. This previously reported finding suggests that deficits in ADHD and CTD may simply add-up in children with co-existing ADHD+CTD, suggesting that ADHD+CTD may represent a simple combination of both disorders and no distinct clinical entity [18]. This leads to the question how these comparable difficulties on the performance level may arise.

As expected from the retinal dopaminergic hypothesis [15], difficulties in patients were more pronounced on the blue-yellow axis, which was clearly not the case in controls. This finding was recently supported by a study of early colour visual evoked potentials, in which children with ADHD compared to Controls showed enhanced P1 amplitude during blue and yellow processing, whilst red and green and achromatic stimuli evoked a P1 that did not differ from Controls [48]. Since that study incorporated easily discriminable sinusoidal wave gratings, larger P1 amplitudes evoked by blue and yellow stimuli may index compensatory activity in ADHD which may fail in a more difficult discrimination test leading to particular difficulties on the blue-yellow axis detected in the current study.

The current results also agree partly with findings in adults with ADHD, which showed higher total error scores in the FMT and particularly pronounced deficits along the blue spectrum, but with considerably lower (medium) effect size that detected in this study with children [16]. One may speculate whether both findings may be driven by a transient developmental lag within the dopaminergic system. However, colour perception deficits were unrelated to general cognitive ability as indicated by IQ, so it remains open for further research in which way colour discrimination taps a specific or distinct part of dopaminergic processing.

### Sustained attention

The three main outcome variables of the FAIR [44] revealed attention problems in children with ADHD but not CTD, following an additive model, whereby children with ADHD+CTD showed very similar impairments as children with pure ADHD (see Fig 2 for the FAIR-L score reflecting the number of items processed in 6 minutes and Table A in S1 File). This outcome regarding ADHD is in agreement with a study on FAIR performance in children and adolescents with different psychiatric disorders, suggesting that ADHD was particularly associated with difficulties in Q and K, but only mild problems in L [49]. Moreover, false alarms were more frequent in ADHD, possibly suggesting a more impulsive response style. Children with pure CTD showed numerically but not significantly (all p>.2) lower scores than Controls, possibly reflecting small effects that remain undetected in the current study. These results are in line with previous studies, indicating that cognitive deficits in the comorbidity of ADHD+CTD may be mostly driven by ADHD, following an additive model [10]. However, attention deficits in ADHD may be part of a more general cognitive deficit, as these specific findings disappear when controlling for IQ.

### Interference liability

Deficits with Cognitive Control in ADHD are a frequently reported finding in ADHD, and early cognitive theories suggest that in particular behavioural inhibition may be “the” core deficit of ADHD [21]. This view is challenged by a number of findings, suggesting that ADHD is a neuropsychologically heterogeneous disorder [50], and deficits in executive functions may neither be specific nor a sufficient precondition [51, 52].

The current single-trial Stroop-Tests differentiate speed and accuracy as two aspects of task performance. The data revealed large Congruency effects in response speed and accuracy, for both Colour- and Counting-Stroops. **Response speed** was generally slower in children with CTD, but no considerable differences regarding interference liability between groups were detected. On the other hand, important effects regarding **accuracy** were found for children with ADHD who performed less accurate in both Stroops. Besides these general accuracy effects, the Colour Stroop revealed larger Congruency effects on accuracy in both pure ADHD and CTD groups, whilst the Counting-Stroop did not. Importantly, this divergent finding was driven by larger congruency effects in Controls and comorbid ADHD+CTD, whilst both pure ADHD and CTD groups accuracy remained unchanged. One may speculate whether differences between colour naming and counting on the one hand and the mapping of the response to the respective button might have played a role.

The **combined analysis of both speed and accuracy** in one statistical model revealed several important effects. First, in the Colour Stroop, children with ADHD showed (compared to controls) a significant shift towards response speed on the expense of accuracy, whilst in comparison children with CTD and also those with ADHD+CTD showed a shift towards more accurate responses. Hence, response style may be an important neuropsychological difference between ADHD (i.e. speed) and CTD (i.e. accuracy). However, as the current Stroop implementation revealed differences between groups not in response speed, but rather accuracy, these combined speed-accuracy analyses merely reflect accuracy findings. The missing of such differential trade-offs in the Counting Stroop must remain unexplained by our data.

#### The role of colour perception in interference liability

Since at least the Colour-Stroop relies on colour processing, performance may be moderated by colour discrimination, and deficits as detected here in ADHD and CTD may compromise the interpretation. The current analysis of covariation indicated that colour discrimination had an unspecific impact on Stroop performance and may partly explain these unspecific performance problems in children with ADHD. A more recent study with adults suggest that exogenous covert attention (modulated by cue presentation) may has an impact on colour saturation discrimination, but this may not explain impairments in ADHD [53]. But as reported in previous studies with the classical Colour-Stroop procedure, its impact on the congruency effect is probably negligible [17, 18]. It does also not explain the divergent interference effects detected with the Colour- and Counting-Stroop. Moreover, Stroop performance was unrelated to inter-individual differences in general cognitive ability as measured with IQ.

### Limitations

The current study has limited statistical power, and as such potentially relevant small to medium sized effects may remain undetected. However, the detected attention difficulties in ADHD, additive colour discrimination deficits in ADHD and CTD, as well as the sub-additive effects on interference load in the Colour-Stroop, were in fact of medium to large size, suggesting that sample size was at least adequate for testing the current hypotheses, but a larger sample would nevertheless mean a stronger empirical basis for the interpretation of the results. Further comorbidities, with the exception of severe psychiatric (e.g. Autism Spectrum Disorder) or neurologic disorders that may mimic ADHD or CTD, were not considered in the current study. Another important limitation is our limited understanding of the equivocal interference effects, namely that Controls and children with ADHD+CTD showed difficulties in the Counting- that were not present in the Colour-Stroop, and the role of colour perception thereon. To our understanding, this predicament requires further research particularly on underlying brain activity before definitive conclusions can be drawn.

## Conclusion

The current study further elucidated the impact of ADHD and CTD and their co-existence on neuropsychological functions. The co-existence of ADHD and CTD may thus be characterized as a hybrid: Attention problems may be a part of lower general cognitive ability in ADHD and may be not a major issue in pure CTD. Colour discrimination deficits associated with dopaminergic dysfunctions may be independently associated with both ADHD and CTD, and may thus be particularly expressed in co-existing ADHD+CTD, following an additive model. Interference control assessed with parallel Stroop-Tests yielded equivocal effects that may not be clarified by colour discrimination deficits: while the Colour-Stroop showed impairments in children with ADHD and CTD, but not in comorbid ADHD+CTD, the parallel Counting-Stroop did not show performance deficits in patients.

Taken together, ADHD and CTD are associated with neuropsychological deficits probably related to dopaminergic dysfunctions. Attention deficits were primarily associated with ADHD while colour discrimination deficits were driven by ADHD and CTD following an additive model. Equivocal findings regarding interference control may indicate limited validity of the Stroop-Paradigm for clinical assessments.

## Supporting information

S1 File Supplementary material. Table A gives mean scores and standard deviations of spelling ability and word fluency, Farnsworth-Munsell 100 hue error scores and scores from the FAIR attention test. Table B gives performance data (mean RT of correct responses and error-rates) from the Colour- and Counting-Stroop. Figure A gives example of the Frankfurt Attention Inventory (FAIR). Figure B illustrates the Stroop Response-Pad layout. Figure C gives confidence intervals from the analysis of inter-individual speed-accuracy tradeoff.
(PDF)
