# Supplementary material for: Revisiting the co-existence of Attention-Deficit/Hyperactivity Disorder and Chronic Tic Disorder in childhood—The case of colour discrimination, sustained attention and interference control
Source: PLoS One. 2017 Jun 8;12(6):e0178866. doi: 10.1371/journal.pone.0178866 (PMC5464598; doi:10.1371/journal.pone.0178866)
Supplement: S1 File — Table A gives mean scores and standard deviations of spelling ability and word fluency, Farnsworth-Munsell 100 hue error scores and scores from the FAIR attention test. Table B gives performance data (mean RT of correct responses and error-rates) from the Colour- and Counting-Stroop. Figure A gives example of the Frankfurt Attention Inventory (FAIR). Figure B illustrates the Stroop Response-Pad layout. Figure C gives confidence intervals from the analysis of inter-individual speed-accuracy tradeoff. (PDF) [file pone.0178866.s001.pdf]

Table A: Dyslexia, Color Perception & Sustained Attention

Measure	Controls (C) Mean (SD)	ADHD (A) Mean (SD)	CTD (T) Mean (SD)	ADHD+CTD (AT) Mean (SD)	ANOVA $F_{(1,65)}$ - (part. η2) ADHD	CTD	ADHD*CTD
**Possible Dyslexia (N)**[1]	2	3	2	1		$\chi^2(1)=2.1$, p=.55	
**Spelling Abilities (T score)**	50.6 (12.3)	46.1 (9.2)	48.7 (7.5)	46.7 (7.8)	2.0 (.03)	<1 (<.01)	<1
**Word Fluency (n/3 min)**	25.7 (7.2)	27.2 (11.4)	22.8 (5.5)	24.4 (9.7)	<1	1.9 (.03)	<1
**Farnsworth-Munsell 100 hue (error score)**							
Total	52.4 (28.1)	85.6 (33.3)	98.0 (35.9)	156.1 (51.6)	22.6** (.26)	36.5** (.36)	1.7 (.03)
blue-yellow	27.1 (17.5)	49.0 (21.8)	53.6 (25.0)	86.5 (29.8)	20.9** (.24)	28.5** (.31)	0.8 (.01)
red-green	25.3 (11.8)	36.6 (15.2)	44.4 (19.3)	69.6 (26.8)	14.2** (.18)	28.7** (.31)	2.1 (.03)
Difference	1.7 (9.9)	12.4 (17.4)	9.3 (26.6)	17.0 (23.5)	3.1+ (.05)	1.4 (.02)	0.1 (<.01)
**FAIR**[2]							
L	262 (83)	218 (68)	239 (56)	219 (68)	3.6* (.05)	<1	<1
Q	.91 (.06)	.86 (.08)	.90 (.08)	.86 (.07)	4.8* (.07)	<1	<1
K	239 (83)	191 (81)	216 (58)	190 (63)	4.7* (.07)	<1	<1
False Alarms	2.8 (1.4)	6.4 (1.5)	4.2 (1.2)	6.2 (1.2)	4.2* (.06)	<1	<1

[1] Dyslexia diagnosis requires the Spelling T-score ≤40 and at least 15 T-points below the (T-transformed) IQ. This information is missing for one subject with ADHD+CTD.
[2] Frankfurter Aufmerksamkeits Inventar: number (L) and proportion (Q) of concentrated processed items, continuity (K)
$^{+}$ p<.1, * p<.05, ** p<.01

Table B: Stroop-Test Performance

Measure	Controls (C) Mean (SD)	ADHD (A) Mean (SD)	CTD (T) Mean (SD)	ADHD+CTD (AT) Mean (SD)	ANOVA $F_{(1,65)}$ - (part. η2) ADHD	CTD	ADHD*CTD
**Single Trial Stroop-Tests**							
**RT (ms)**							
**Color-Stroop**							
Color/Bar	648 (104)	655 (93)	711 (120)	791 (186)	1.7 (.03)	9.1** (.12)	1.2 (.02)
Color/Word	709 (143)	727 (116)	798 (140)	847 (174)	0.8 (.01)	8.5** (.12)	<1 (<.01)
Difference	61 (47)	72 (48)	87 (79)	56 (117)	<1 (<.01)	<1 (<.01)	1.1 (.02)
**Counting-Stroop**							
Number/Dots	654 (99)	675 (66)	684 (116)	762 (151)	3.0+ (.04)	4.2* (.06)	1.0 (.02)
Number/Digits	733 (138)	764 (80)	767 (137)	839 (160)	2.4 (.04)	2.8+ (.04)	<1 (<.01)
Difference	78 (78)	88 (69)	83 (68)	76 (74)	<1 (<.01)	<1 (<.01)	<1 (<.01)
**Accuracy (% correct)**							
**Color-Stroop**							
Color/Bar	90 (10)	81 (11)	93 (4)	88 (12)	8.7** (.12)	4.7* (.07)	0.5 (<.01)
Color/Word	89 (7)	71 (15)	85 (10)	84 (11)	12.7** (.16)	3.5+ (.05)	10.4** (.14)
Difference	1 (4)	10 (12)	8 (9)	3 (9)	1.4 (.02)	<1 (<.01)	9.9** (.13)
**Counting-Stroop**							
Number/Dots	91 (8)	87 (10)	92 (7)	87 (12)	3.9+ (.06)	<1 (<.01)	<1 (<.01)
Number/Digits	83 (10)	79 (8)	86 (9)	76 (14)	6.5* (.09)	<1 (<.01)	1.6 (.02)
Difference	9 (9)	8 (10)	7 (7)	12 (15)	<1 (.01)	<1 (<.01)	1.0 (.02)

$^{+}$ p<.1, * p<.05, ** p<.01

Figure A: Example of the Frankfurt Attention Inventory (FAIR)

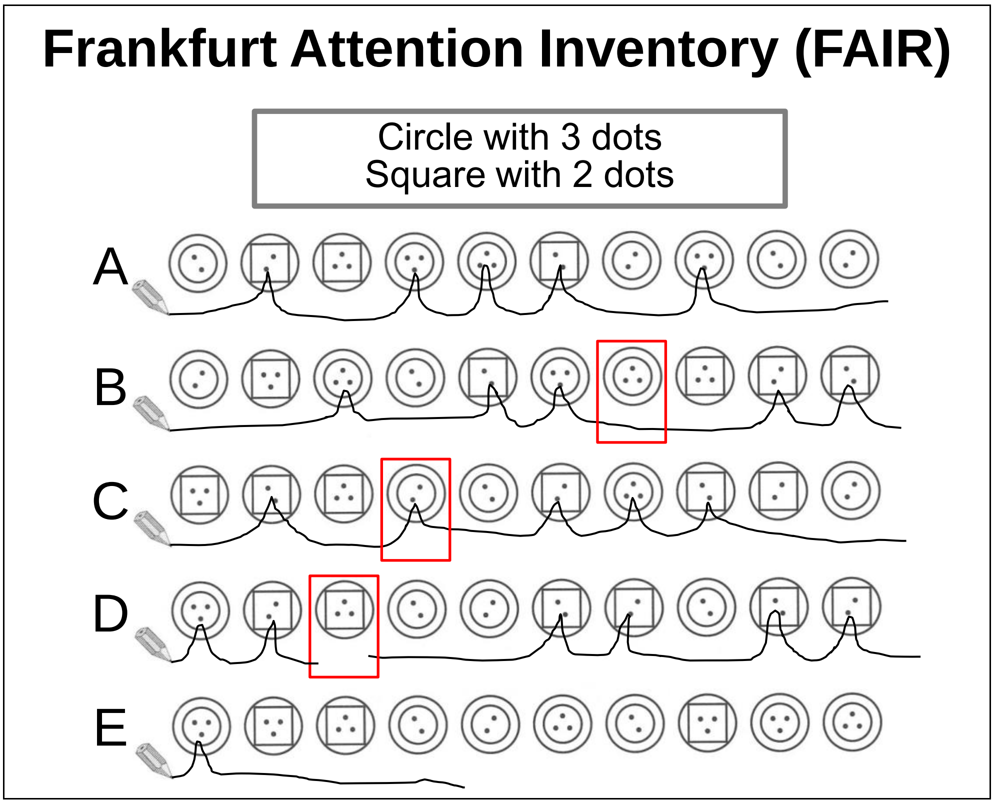

The paper and pencil FAIR requires continuously marking items, arranged in rows with 20 each during a 6 min assessment. The example gives 10 items per row where the targets (here a circle with 3 dots or a square with 2 dots) require a spike while the non-targets need to be underlined, all without stop of the drawing. Row A shows correct processing; omission error (B), false alarm (C) and line error (D) are marked red, in line E the last processed item is #4. The main performance parameters L (indicating the error-corrected estimate of concentrated processed number of items), Q (quality or accurateness, the proportion of L amongst the total number of processed items) and K (the product of Q*L as a continuity indicator):

L = [number of total items processed]-[line errors]-2*[omission errors+false alarms]

Q = L/[number of total items processed]

K = L*Q

More details are given in Moosbrugger & Oelschlägel, 1996.

Figure B: Stroop Response-Pad layout

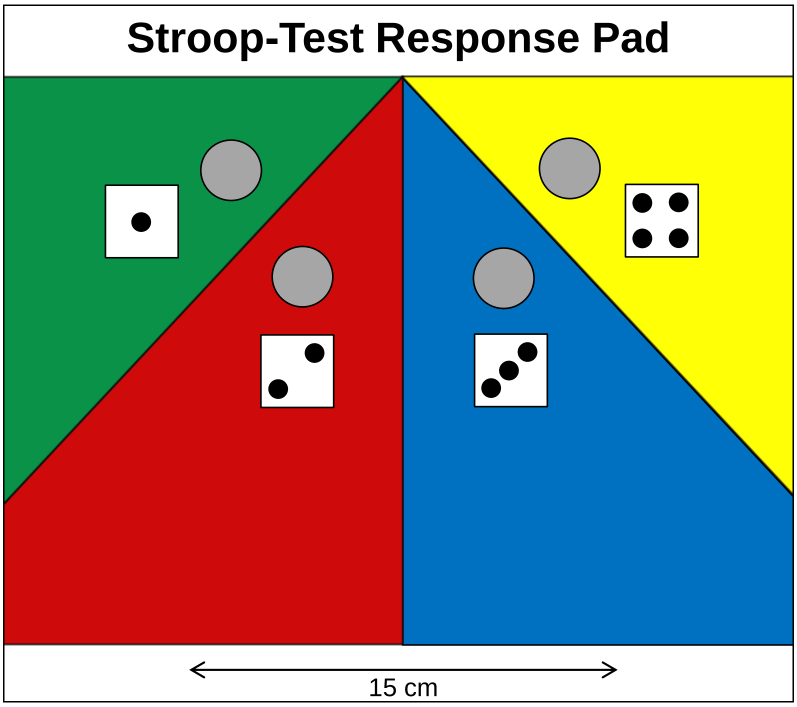

Schematic illustration of the Response-Pad used for both Color- and Counting-Stroop. The response buttons (grey circles) were associated with the four target colors and the four quantities.

Figure C: Speed-Accuracy tradeoff in ADHD and CTD

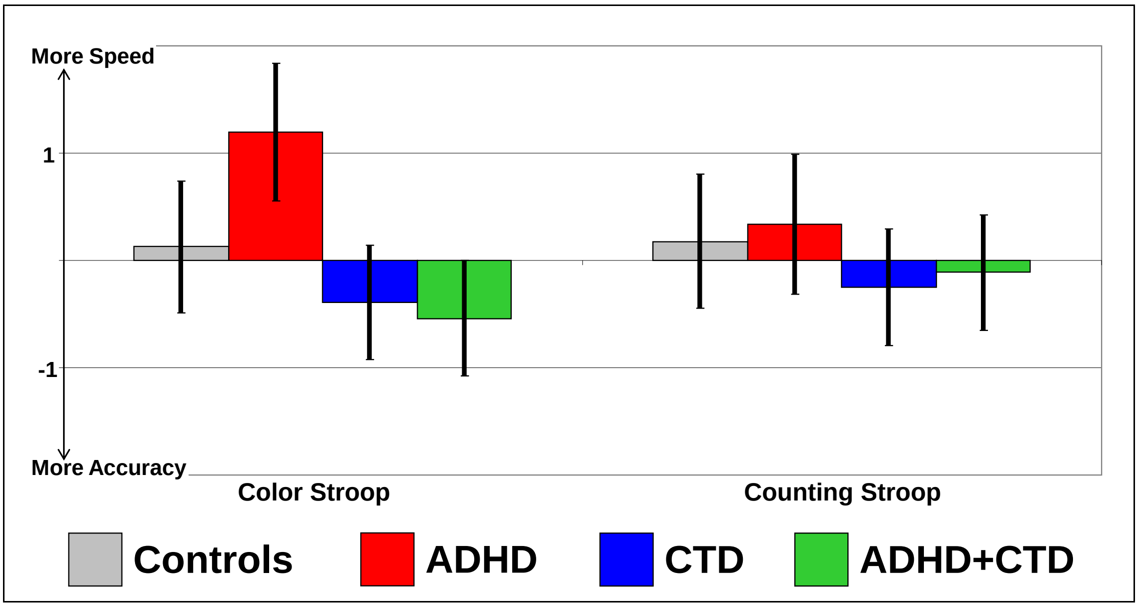

Analysis of inter-individual speed-accuracy tradeoff (as the mean difference between z-transformed reaction-times minus z-transformed accuracy, presented with confidence intervals, p=.05). The Color-Stroop (left) revealed compared to Controls (grey), a significant emphasis on speed in ADHD (red) and a shift towards accuracy in children with comorbid ADHD+CTD (green), also as a tendency (p=.10) present in CTD (blue). In contrast, no such tradeoff group-differences were significant in the Counting-Stroop (all CIs overlap).
